# Bilateral Carotid and Vertebral Artery Dissection from Blunt Trauma

**DOI:** 10.1155/2018/1919034

**Published:** 2018-03-12

**Authors:** Catherine Coss, Jeffrey Jones

**Affiliations:** Spectrum Health, Michigan State University Emergency Medicine Residency Program, Grand Rapids, MI, USA

## Abstract

Carotid and vertebral artery injuries are rare following blunt trauma. They can, however, lead to severe consequences with a significant associated rate of stroke and intracranial hemorrhage, particularly if the diagnosis and treatment are delayed. We report a case of a 23-year-old female who presented to the Emergency Department with bilateral carotid and vertebral artery dissection following a motor vehicle collision (MVC).

## 1. Introduction

Cerebrovascular injury is readily part of the differential diagnosis in a penetrating neck trauma, but approximately 1% of blunt trauma also has associated cerebrovascular injury [1–6]. These injuries have significant morbidity and mortality, particularly if the diagnosis and the treatment are delayed [5–8]. Patients can present with minimal to no initial neurologic symptoms, so screening is a valuable tool to avoid missing this injury [9, 10]. Internal carotid arteries are more commonly injured than the vertebral arteries [2, 7]. While bilateral injuries are unusual, four vessel blunt cerebrovascular injuries (BCVI) are extremely rare. This case report presents a young female who presented with bilateral carotid and vertebral artery dissection and whose course was further complicated by a rare genetic metabolic disorder.

## 2. Case Presentation

A 23-year-old female patient arrived at the Emergency Department trauma bay following an MVC. She was the restrained driver when she was rear-ended by a truck and slammed forward into a guardrail. Patient was unresponsive at the scene for paramedics and required extrication from the vehicle. Upon arrival in the trauma bay, patient was awake and responsive, repeatedly crying out and answering, “I do not know” and “ok” to all questions. GCS was 14 and no injuries were immediately noted. She was mildly tachycardic with a pulse of 102; her other vital signs were normal. The patient was unable to provide a medical history, but her phone had a message on it stating that she had holocarboxylase synthetase deficiency. When parents arrived, they confirmed this and stated that she had a history of Asperger's syndrome.

CT scan of the patient's cervical spine was performed with contrast, and she was found to have bilateral internal carotid and vertebral artery dissections without signs of bony injury. Subsequent MRI of the brain showed acute infarcts of bilateral cerebral hemispheres, greater on the left than on the right, and a right-sided cerebellar infarct, without signs of cervical spine ligamentous injury. CT angiography showed dissection of the left internal carotid starting at the level of C2, dissection of the right internal carotid originating at the bifurcation, and bilateral vertebral dissection at the level of C2-C3, with significant thrombosis present in the vertebral arteries.

The patient developed worsening mental status and became unresponsive to stimuli, though she was able to maintain her airway. She would still intermittently spontaneously move her left side but had little to no movement of the right upper and lower extremities and had a leftward deviated gaze. Heparin drip and aspirin were started. Patient was not considered a candidate for endovascular intervention since she had no focal areas that could be addressed operatively. By the subsequent day, patient had developed a significant lactic acidosis, with a bicarbonate of 8 mmol/L, an anion gap of 26 mmol/L, and a lactate of 6.2 mmol/L. This was likely secondary to the patient's underlying genetic metabolic disorder. Patient was started on a bicarbonate drip. Neurology was consulted and a continuous EEG was obtained, which showed areas of decreased activity without signs of seizure. A nasointestinal tube was placed and the patient's home regimen of biotin and carnitine was able to be resumed. The lactic acidosis was corrected, and the bicarb drip was stopped.

On day 3, patient was able to transition out of the ICU, though she was still not responding to commands or interacting in any way. Two days later she was transitioned from the heparin drip to an enoxaparin bridge to warfarin. Serial CT scans of the head revealed evolving infarct without signs of hemorrhagic conversion. By day 6, the patient's neurologic status was slightly improving. She was able to answer yes/no questions and state her name. On the day of discharge two days later, repeat CT angio of the head and neck showed improvement of the vascular function and the patient was able to follow simple commands and walk during physical therapy. She was discharged to acute rehab on warfarin, biotin, and carnitine.

## 3. Discussion

There has been increasing recognition of injury to the cerebrovascular vessels in the context of blunt trauma. The incidence has been reported as low as 0.1% in blunt trauma, but more recent literature has indicated an incidence greater than 1% [1–6]. Possibly this increase is due to the expanding use of screening computed tomographic angiography (CTA) to identify vascular injury [2, 3]. The most common etiology of traumatic cerebrovascular injury is motor vehicle accidents [1, 2, 7, 11], with the highest risk factor being motorcycle crash [4]. Other potential causes include near hanging, chiropractic manipulation, falls, assault, or iatrogenic injury [8, 10, 12]. The pathophysiology differs slightly for injury to the internal carotids versus the vertebral arteries, though both result from pathology to the cervical spine. The internal carotid arteries are generally injured through flexion-distraction or compression, hyperextension, hyperrotation, or direct impact [11, 13]. The main mechanism by which the vertebral arteries are injured is hyperextension [11].

There has been much debate about the utility of screening for cerebrovascular injury in blunt trauma patients. Many different screening criteria have been discussed, such as the Denver screening criteria, which include physical exam findings and risk factors [14]. Both the Western Trauma Association and the Eastern Association for the Surgery of Trauma have published guidelines to help determine which patients should be screened and have distinct criteria for which patients CTA should be obtained [6, 13]. In our patient, however, she would not have qualified for screening by any of the proposed criteria or guidelines, since her BVCI was her only trauma related injury. At our institution, CT of the cervical spine with contrast is routinely obtained for all trauma patients with a significant mechanism, which is how her injury came to be found. Multiple studies and review have found that CTA is an effective screening tool if it is equal to or greater than 8-slice multidetector array, with a sensitivity of 100% for carotid injury and 96% for vertebral injury [13, 15]. Ultrasound was found to be ineffective with a sensitivity of 38% [11]. Angiography is still technically the gold standard but is invasive and carries its own risk of vascular injury, so it is now only recommended if clinical suspicion is high and there is a negative CTA [5, 6, 11].

The diagnosis of BVCI can be difficult to make. The symptom presentation can often be delayed [9, 10] and is highly variable depending on the location of the injury and if there is subsequent stroke. Symptoms include asymptomatic, ipsilateral headache, neck pain, facial pain, cranial nerve palsies, Horner syndrome, dizziness, vomiting, reduced GCS, or many different focal neurologic deficits [9–11]. There is also a difference between symptoms in carotid versus vertebral injury. Carotid injury tends to appear similar to a cerebral stoke with cranial nerve deficits, hemiplegias, and altered mental status [8]. Vertebral artery injuries more commonly present as a posterior stroke with vertigo, nausea, vomiting, ataxia, and altered mental status [11]. There have been multiple studies to determine risk factors for BVCI. The consensus is that carotid injuries are most likely with mandible or facial fractures and vertebral injuries with cervical spine fractures and both are more likely with decreased GCS, increased injury severity scores, or basilar skull fractures [5, 12]. Our patient demonstrates the difficulty of diagnosing BVCI. She was initially asymptomatic, though it could be argued that her GCS of 14 was an early indicator. It was not until later when she developed bilateral strokes that her symptoms of severely altered mental status, right hemiplegia, and leftward deviated gaze appeared. This emphasizes the importance of screening in these patients.

Early implementation of anticoagulation to prevent stroke is the mainstay of treatment in BVCI [3, 6, 8, 11, 13–15]. The most commonly used agent is heparin, followed by transition to warfarin, though there has been evidence that antiplatelet therapy with clopidogrel and/or aspirin may be equally effective [6, 11, 13, 14]. Surgery is not recommended unless there is complete transection of the vessel or early neurologic deficit with an accessible lesion [6, 11, 13]. There is more debate about the advisability of endovascular intervention in these cases. Most of the literature agrees that stents should not be placed initially due to the increased risk of bleeding [13, 15, 16]. Cothren et al. found that carotid artery stents placed for pseudoaneurysm of BVCI had a 21% complication rate and a 45% occlusion rate [16]. Both the Western Trauma Association and the Eastern Association for the Surgery of Trauma, however, recommend repeat CTA 7–10 days after trauma and placing stents if a pseudoaneurysm has developed or if there is significant narrowing of the vessel [6, 13]. There is no consensus about the length of time necessary to continue anticoagulation, as there have been no good studies addressing this specific issue [6, 13].

The incidence of all four vessels injured in BVCI is incredibly rare. There are only 6 published cases for four vessel BVCI [9, 15, 17–20]. This patient's case is even more unique in that her four-vessel BVCI was her only traumatic injury and her medical course was complicated by a rare metabolic disorder. There is no indication in the literature that holocarboxylase synthetase deficiency contributed to her vascular injuries. She was started on heparin early but still underwent a massive bilateral stroke. Her recovery was significant however; and at a 1-month followup her only deficits were mild speech difficulties, right quadrantanopia on her vision, and mild right-sided weakness.

In summary, early recognition and treatment of BCVIs have been shown to prevent stroke-related morbidity and mortality. Early screening of asymptomatic patients based on injury-related mechanism and/or associated injuries is recommended to identify the at-risk population. The goal in managing vascular dissection is to prevent neurologic deficits. Because thromboembolic disease is the most common cause of ischemia in these cases, treatment includes anticoagulation and antiplatelet therapy.
